# *Plasmodium vivax* malaria across South America: management guidelines and their quality assessment

**DOI:** 10.1590/0037-8682-0179-2020

**Published:** 2020-10-05

**Authors:** José Alejandro Iza Rodríguez, Shirley Natali Iza Rodríguez, Mario Javier Olivera

**Affiliations:** 1 Universidad Nacional de Colombia, Department of medicine, Bogotá, Colombia.; 2 Universidad Militar Nueva Granada, Department of Medicine, Bogotá, Colombia.; 3 Instituto Nacional de Salud, Parasitology Group, Bogotá, Colombia.

**Keywords:** Plasmodium vivax, Antimalarials, Clinical practice guidelines, AGREE

## Abstract

**INTRODUCTION:**

*Plasmodium vivax* malaria represents a major public health problem. This study presents the quality assessment of clinical practice guidelines for the management of *P. vivax* malaria.

**METHODS:**

A systematic review was conducted in PubMed, SciELO, and Google Scholar. Additionally, five guidelines were assessed with the AGREE (Appraisal of Guidelines Research and Evaluation) II protocol.

**RESULTS:**

The general performance on the domains of stakeholder involvement, development rigor, and editorial independence was low.

**CONCLUSIONS:**

Most guidelines lack a solid research methodology, which implies ambiguous accuracy. Much needs to be done in the area of therapeutics and quality of policies.

Malaria infections cause major morbidity and mortality, representing a vast global health issue. The majority of malaria cases in the Americas are caused by *Plasmodium vivax* over *Plasmodium falciparum* ^1^; nevertheless, in the Colombian Pacific coast, the proportion is reversed^2^. Despite the sustained downward trend of malaria rates from 2000-2014 in the Americas, an overall rise has been recently observed, largely in Venezuela and, to a lower degree, countries such as Brazil, Guyana, Colombia, and Ecuador. This contrasts with the rates in Paraguay and Argentina, which were certified by the WHO in 2018 and 2019, respectively, for the elimination of malaria^1^ ^,^ ^2^. In comparison with *P. falciparum, P. vivax* shows less virulence but hides in the host for several months and even years in its dormant liver stage, which can cause relapses, a characteristic that shows evolutionary efficiency developed over time^3^. 

*P. vivax* malaria has been considered a benign infection, but some literature dismisses the “benign” notion since there have been reported presentations of severe anemia and organ dysfunction^4^. The spectrum of disease caused by *P. vivax* is modulated by host susceptibility and response, age of exposure, and various parasite factors such as antimalarial drug resistance^3^ ^,^ ^4^. For decades, chloroquine (CQ) has been the mainstay of treatment with outstanding effectiveness against asexual stages in the peripheral blood, but no activity against hypnozoites; thus, the standard recommendation to prevent future relapses is the simultaneous or sequential treatment with primaquine (PQ) in appropriate doses^5^. 

The persistence of asexual *P. vivax* in the blood during the follow-up period in the presence of therapeutic concentrations of CQ defines treatment failure due to resistance, which was first documented in 1989 in Papua New Guinea^5^. Since then, sporadic strains resistant to CQ have been reported in other countries, including Colombia^6^, Brazil^2^ ^,^ ^7^ and Guyana^8^, and a worrying prevalence in South East Asia is emerging^1^. Little is known about the exact mode of action of CQ or the resistance mechanism in *P. vivax*, since continuous culture of this species is not feasible^9^. Regarding the mechanism of action of CQ, most *in vitro* cultures and studies have used *P. falciparum* as a model, suggesting that the heme polymerase is the target molecule. Data from *in vitro* CQ sensitivity studies in *P. vivax* report stage-specific effects of CQ, which raises more questions about antimalarial pharmacodynamics in *P. vivax* malaria, highlights the differences between these malaria species^3^ ^,^ ^9^, and calls for the formulation of strategies to avoid the belief that established protocols cause a delayed response to resistance when CQ efficacy collapses.

Despite this reported resistance, CQ is an acceptable first-line therapy in guidelines in South America for CQ-sensitive *P. vivax*, given its low cost and wide availability, which are major factors that determine the use of antimalarial drugs in most endemic countries with limited resources^5^. 

PQ is the only drug licensed, with proven efficacy, to eliminate the dormant stages of *P. vivax* and *Plasmodium ovale.* It is always used in combination with a schizontocidal agent, except if used as a primary prophylaxis, for which its indication is not yet licensed due to the limited trial data, side effects, and cost effectiveness of the intervention vs cost of screening for G6PD deficiency^10^. Trials demonstrated adequate tolerance in therapeutic doses, but other complications have also been described aside from hemolysis in G6PD-deficient patients, such as hemolysis in non-G6PD-deficient individuals, mild or self-limited methemoglobinemia, gastrointestinal complaints, and allergies^11^. True resistance to PQ is difficult to establish apart from the associated agent; moreover, noncompliance that is secondary to the long duration of therapy and reinfection in malaria endemic regions raises doubts about resistant strains. In areas with a low prevalence of G6PD deficiency or other complications associated with PQ administration, higher doses or longer courses may improve efficacy^10^ ^,^ ^11^.

Therapeutic regimens for *P. vivax* indicate the administration of two or more drugs, as a single drug scheme targets only specific stages of the parasite (Table 1).

**TABLE 1:** Treatment policy for *Plasmodium vivax* malaria.

		Colombia	Venezuela	Brazil	Peru	Ecuador*	Guyana
Uncomplicated	1st line	**CQ**	**CQ**	**CQ**	**CQ**	**CQ**	**CQ**
		d1: 10 mg/kg;	d1: 10 mg/kg;	d1: 10 mg/kg;	d1: 10 mg/kg;	d1: 10 mg/kg;	d1: 10 mg/kg;
		d2: 7.5 mg/kg;	d2: 10 mg/kg;	d2: 7.5 mg/kg;	d2: 10 mg/kg;	d2: 7.5 mg/kg;	d2: 7.5 mg/kg;
		d3: 7.5 mg/kg.	d3: 5 mg/kg.	d3: 7.5 mg/kg.	d3: 5 mg/kg.	d3: 7.5 mg/kg.	d3: 7.5 mg/kg
		**PQ**	**PQ**	**PQ**	**PQ**	**PQ**	**PQ**
		0.25 mg/kg/d for 14 d	0.25 mg/kg/d for 14 d	0.5 mg/kg/d for 7 d	0.5 mg/kg/d for 7 d	0.5 mg/kg/d for 14 d	0.25 mg/kg/d for 14 d
	2nd line/relapse	**CQ**	**CQ**	**AL**	-	**CQ**	**AL**
		d1: 10 mg/kg;	d1: 10 mg/kg;	80-480 mg/12 h		d1: 10 mg/kg;	80-480 mg/12 h
		d2: 7.5 mg/kg;	d2: 10 mg/kg;	for 3 d		d2: 7.5 mg/kg;	for 3 d
		d3: 7.5 mg/kg.	d3: 5 mg/kg.	**PQ**		d3: 7.5 mg/kg.	PQ
		**PQ**	**PQ**	0.5 mg/kg/d for		**PQ**	0.5 mg/kg/d for
		0.25 mg/kg/d for 14 d	0.25 mg/kg/d for 14 d	14 d		0.5 mg/kg/d for 14 d	14 d
Severe	1st line	**AS**	**AS**	**AS**	**AS**	**AS**	**AS**
		2.4 mg/kg at 012, and 24 h, then daily for 7 d.,	2.4 mg/kg at 012, and 24 h,, then daily for 7 d,	2.4 mg/kg at 012, and 24 h, then daily for 7 d.,	2.4 mg/kg at 012, and 24 h, then daily for 7 d. ,	2.4 mg/kg at 012, and 24 h, then daily for 7 d.,	2.4 mg/kg at 012, and 24 h, then daily for 7 d.,
					**Cdm**		
					10 mg/kg/12 h for 7 d		
	2nd line	**Quinine**	**AR IM**	**Cdm**	**Quinine** bolus	**AR IM**	**AR IM**
		bolus 20 mg/kg; then 10 mg/kg/8 h for 7 d.	bolus 3.2 mg/kg; then 1.6 mg/kg/d for 5 d.	10 mg/kg/8 h for 7 d.	20 mg/kg; then 10 mg/kg/8 h for 48 h	bolus 3.2 mk/kg; then 1.6 mg/kg/d for 5 d.	bolus 3.2 mg/kg; then 1.6 mg/kg/d for 5 d.
		**Cdm**			**Cdm**		
		10 mg/kg/8 h for 5 d.			10 mg/kg/12 h		

**CQ:** chloroquine; **PQ:** primaquine; **AS:** artesunate; **AL:** artemether lumefantrine; **AR:** artemether; **Cdm:** clindamycin. * Malaria diagnosis and treatment protocol 2019^12^.

A first-line therapeutic strategy in uncomplicated malaria includes a combination of CQ plus PQ, with slight differences only in the dosage and duration of PQ (the highest being in Ecuador with a dosage of 14 mg/day for 14 days, within the safe therapeutic range). Colombia, Venezuela, and Ecuador insist in the repeated administration of CQ plus PQ in case of initial treatment failure, due in part to a complete remission with this combination^6^ ^,^ ^12^ ^,^ ^13^ (no evidence of resistance to those medications). However alternative second-line treatments recommended in Brazil and Guyana are equally effective and reveal the presence of resistance to CQ in some parts of the Amazon region^2^ ^,^ ^7^ ^,^ ^8^. 

Regarding therapeutic schemes in severe *P. vivax* malaria, the same approach is made as in severe malaria caused by *P. falciparum*. Second-line schemes range from Quinine plus Clindamycin, Artemether, or even Clindamycin alone depending on the availability of medications per medical center as stated in the Brazilian guideline. 

The use of clinical practice guidelines (CPG) is essential for upgrading and unifying diagnostic and therapeutic approaches in order to have an impact on public health indices such as mortality and disease prevalence. Hence, a thorough assessment of their quality is relevant to improve the development of CPG. 

An electronic study of the current national malaria guidelines (published between 2010-2019) was performed on official websites of the public health institutes and ministries of Colombia, Venezuela, Brazil, Peru, and Guyana. For Ecuador, no official CPG was found.

The AGREE (Appraisal of Guidelines Research and Evaluation) II validation instrument was applied to these guidelines for a standard evaluation. For a publication to be included, it had to be a CPG; manuals and protocols were not considered, and thus the Ecuador protocol was not included in the assessment. Two appraisers followed instructions from the AGREE II user’s manual. Any disagreement was resolved by a third reviewer. Agreement between reviewers was calculated using the kappa coefficient (κ) and was interpreted based on the approach by Landis and Koch. Statistical analyses were performed using Stata® v14.0 (Stata, College Station, TX). The overall agreement between the reviewers was high: κ = 0.92 (95% confidence interval: 0.91-0.93; Table 2).

**TABLE 2:** Score of each CPG using the AGREE II validation instrument

Guidelines	Scope/purpose	Stakeholder	Rigor of	Clarity of	Applicability	Editorial
	(%)	involvement (%)	development (%)	presentation (%)	(%)	independence (%)
Comprehensive clinical practice guideline for medical care of patients with malaria (Colombia, 2010) ^6^	83	33	55	97	79	16
Treatment guideline in malaria cases (Venezuela, 2017) ^13^	86	41	38	94	79	16
Guideline for the treatment of malaria in Brazil (Brazil, 2019) ^7^	83	41	29	97	81	14
Technical standard for health care of malaria in Peru (Peru, 2015) ^14^	88	47	27	73	68	14
Malaria Treatment Guideline for health facilities in Guyana (Guyana, 2015) ^15^	58	27	29	78	70	14
Median (95% confidence interval)	79.6	37.8	35.6	87.8	75.4	14.8
	(77.2-82.1)	(36.2-39.3)	(33.3-37.9)	(85.5-90.1)	(74.3-76.6)	(14.5-15.1)

A secondary search was conducted in PubMed, SciELO, Google Scholar, and LILACS regarding *P. vivax* epidemiology, therapeutic schemes, and resistance to first-line treatment in the region to contextualize the evaluation of guidelines. 

The main general flaws common to most of the CPGs assessed were poor reports of meticulous methodology in the literature review, and no explicit grading of evidence of their recommendations or information about the cost of its implementation. There were also no guideline reports ranking the confidence of evidence (GRADE system). Furthermore, no guideline reported conflicts of interest, presence of bias, or a clear statement regarding the procedure for updating the CPG. Views and expectations of target populations were not contemplated, so they were not elaborated in the guidelines. The aforementioned factors to improve are part of the AGREE II protocol. 

The Colombian Health Ministry CPG had different management options (1^st^, 2^nd^, and 3^rd^ line). This guideline scored higher than the rest in development rigor because it had a brief description of evidence-based research, and the document itself was a good systematic review. Regarding stakeholder involvement, this guideline failed by not including information about the composition, discipline, and relevant expertise of the development group. Likewise, it has been a decade since it was published, which is a major disadvantage in view of the dynamic epidemiology.

The sources and research strategies in the rest of the guidelines are unknown to the reader.

The Venezuelan guideline had a detailed presentation of their overall objectives and target population. Its good score in stakeholder involvement was due to a thorough exposition of authors and their profession, with an interdisciplinary approach. However, no methodology was mentioned, nor ranking of evidence confidence.

The Brazilian guideline does not mention a methodology or resources for the recommendations, or the grading of evidence, hence their low score in development rigor. On the other hand, it scored the highest in clarity of presentations, for their graphic schemes of medications (including weight, age, and schedule) which are clearly comprehensible. In its favor, it is the most recent of the guidelines cited here (published in 2019). 

The Peruvian Health Ministry document described general and specific objectives in detail, scoring the highest in scope and purpose. However, it scored the lowest in clarity of presentation, since no alternative schemes for *P. vivax* other than a first-line scheme were formulated, which is inconvenient for nonrare cases of relapse or resistance.

The CPG of Guyana did not specifically describe its objectives since they were not listed or labeled. Additionally, there was no reference to the professionals who were involved in its development, other than the minister. Users may not easily find the key points due to its flat and disorganized structure.

Because the average ratings for the six main domains were 55%, the overall quality of the guidelines was moderate. Poor scores in development rigor were consistent, probably in part due to a lack of specific algorithms to search databases. 

Good quality guidelines for *P. vivax* malaria are essential in endemic countries. With some specific modifications, such as complete identification of authors, grading of evidence of recommendations, and a detailed search methodology in all guidelines, these documents can represent a comprehensive approach and help destroy the barriers and limitations of information in all target populations.
